# Both ERK/MAPK and TGF-Beta/Smad Signaling Pathways Play a Role in the Kidney Fibrosis of Diabetic Mice Accelerated by Blood Glucose Fluctuation

**DOI:** 10.1155/2013/463740

**Published:** 2013-07-14

**Authors:** Xiaoyun Cheng, Wenke Gao, Yongyan Dang, Xia Liu, Yujuan Li, Xu Peng, Xiyun Ye

**Affiliations:** ^1^Department of Endocrinology, Shanghai Tenth People's Hospital, Tongji University School of Medicine, Shanghai 200072, China; ^2^Shanghai Key Laboratory of Regulatory Biology, Institute of Biomedical Sciences and School of Life Sciences, East China Normal University, 500 Dongchuan Road, Shanghai 200241, China

## Abstract

*Background*. The notion that diabetic nephropathy is the leading cause of renal fibrosis prompted us to investigate the effects of blood glucose fluctuation (BGF) under high glucose condition on kidney in the mice. *Methods*. The diabetic and BGF animal models were established in this study. Immunohistochemistry, Western blot, and RT-PCR analysis were applied to detect the expression of type I collagen, matrix metalloproteinase-1 (MMP1), metalloproteinase inhibitor 1 (TIMP1), transforming growth factor beta 1 (TGF-**β**1), phosphorylated-ERK, p38, smad2/3, and Akt. *Results*. BGF treatment increased type I collagen synthesis by two times compared with the control. The expression of MMP1 was reduced markedly while TIMP1 synthesis was enhanced after BGF treatment. ERK phosphorylation exhibits a significant increase in the mice treated with BGF. Furthermore, BGF can markedly upregulate TGF-**β**1 expression. The p-smad2 showed 2-fold increases compared with the only diabetic mice. However, p-AKT levels were unchanged after BGF treatment. *Conclusions*. These data demonstrate that BGF can accelerate the trend of kidney fibrosis in diabetic mice by increasing collagen production and inhibiting collagen degradation. Both ERK/MAPK and TGF-**β**/smad signaling pathways seem to play a role in the development of kidney fibrosis accelerated by blood glucose fluctuation.

## 1. Introduction

Diabetes can cause a wide range of health complications such as atherosclerosis, cardiac dysfunction, retinopathy, and nephropathy. Hyperglycemia is a major sign of diabetes mellitus (DM) and the main cause for its various complications. The side effects of hyperglycemia can be categorized into two types, persistent elevation of blood glucose (BG) levels and blood glucose fluctuation (BGF) [1], which are closely correlated with the DM prognosis, pathogenesis, and complications. Larger BGF is associated with a higher incidence of chronic diabetic complications and a poorer prognosis [2]. Clinical studies have shown that the risks associated with long-term BGF were much more detrimental than that of chronic elevation of BG levels [3]. Furthermore, there are some evidences that intermittent fluctuation of high glucose can induce lesions of varying degrees in glomerular mesangial and vascular endothelial cells *in vitro* [4–6]. Thus, the effects of BGF on diabetic complications need to be determined.

Diabetic nephropathy (DN) is the leading cause of renal fibrosis and chronic renal failure [7]. Renal fibrosis was characterized by glomerulosclerosis and tubulointerstitial fibrosis, which would reduce excretory renal function [8]. Although considerable advances have been made to understand that hyperglycemia can promote chronic diabetic nephropathy, there are no published data to describe the effects of BGF under high glucose condition on renal fibrosis.

It is well known that fibroblast proliferation, altered expression and overdeposition of extracellular matrix contribute to progressive diabetic renal fibrosis. Collagen overproduction, as the major contributor of renal fibrosis, is regulated by several fibrogenic factors such as transforming growth factor-beta1 (TGF-*β*1) and matrix metalloproteinases (MMPs). To study the different effects of hyperglycemia and BGF under hyperglycemia conditions on collagen synthesis and degradation in the kidney of diabetic mice, we establish the diabetic and repetitive BGF animal models, respectively.

The inhibition of p38 mitogen-activated protein kinase has been reported to ameliorate renal fibrosis in obstructive nephropathy [9]. TGF-beta is the primary cytokine driving fibrosis in kidney and other organs susceptible to fibrotic injury such as lung and liver. Members of the TGF-*β* superfamily transduce intracellular signals by smad proteins. Smad2 and smad3 act in the TGF-*β*/activin pathway, whereas smad1, smad5, and smad8 are thought to act as bone-morphogenetic-protein- (BMP-) specific smads. Smad2/3 form heteromeric complexes with smad4 and translocate into the nucleus to regulate transcription of target genes. A study demonstrates that TGF-*β* signals can mediate renal fibrosis through smad2/3 [10]. However, the underlying cellular mechanisms by which BGF leads to matrix accumulation in the renal tissue are less well understood.

Therefore, this study investigates whether blood glucose fluctuation in the diabetic mice could accelerate the development and progression of diabetic renal fibrosis *in vivo*. Moreover, we also examine the underlying mechanisms of BGF-associated renal changes to further study the therapeutic potential of inhibiting BGF in diabetic renal fibrosis.

## 2. Methods

### 2.1. Animals

Male Kunming mice (25–28 g) were purchased from Shanghai SLAC Laboratory Animal Co. (Shanghai, China). They were housed in pathogen-free conditions with water and standard mouse chow freely available. Mice were randomly divided into 3 groups (*n* = 10/group): (i) Controls (C), (ii) Diabetic (D), and (iii) Diabetic with BGF (D + BGF).

### 2.2. Diabetic Mouse Model

Animals were fasted overnight and then injected i.v. into the tail vein with 50 mg/kg alloxan that was freshly prepared in normal saline and used within 10 min, whereas controls received saline only. Five days later, mice were fasted for 6 h and then peripheral blood was harvested from the tail vein. Blood glucose levels were determined using a glucose kit and only mice with concentrations between 18 to 21 mM were used for the studies.

### 2.3. BGF Mouse Model

**Figure 1:**
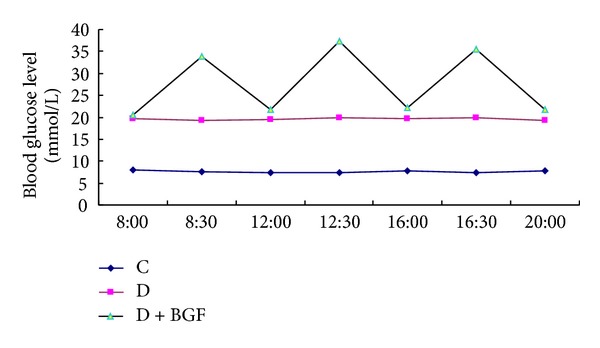
Blood glucose level in the mice at one day after eight-week BGF treatment.

Glucose delivered via i.p. injection was used to establish the BGF model. Diabetic mice were injected i.p. with 2 g/kg of glucose three times a day every 4 h starting at 8 a.m. for 6 weeks. Control and only diabetic mice received saline injections instead of glucose. Blood glucose levels were measured at 7 time points throughout the day for each week to test the regularity of fluctuations as shown in Figure 1. After the animal models were established, ten mice remained in each group for the further experiments.

### 2.4. RT-PCR

After the mice was killed by cervical dislocation, kidney tissue was taken and then weighed for 100 mg. The 100 mg samples were quick-frozen and crushed to powder in a mortar under liquid nitrogen. Total RNA was isolated using Trizol reagent (Invitrogen, USA) according to the instructions of manufacture. RNA samples were quantified by using BioPhotometer (Eppendorf, Germany), and 2 *μ*g total RNA was reverse transcribed into cDNA in 20 *μ*L reaction volume containing 2 *μ*L of random oligonucleotide primer, AMV reverse transcriptase (Invitrogen, USA), and 2 mM dNTP mix. Reverse transcription was performed using a thermal program of 30°C for 10 min, 42°C for 20 min, 99°C for 5 min, and 4°C for 5 min. Then, 1 *μ*L cDNA was used as the templates for PCR amplification in a thermal cycler. The specific primers sequences for human collagen I(*α*2), MMP1, TIMP1, TGF-*β*1, and GAPDH were as provided in Table 1. The cycling program used was 95°C for 10 min followed by 35 cycles of 95°C for 30 s, 58°C for 30 s, and 72°C for 45 s. PCR products were electrophoresed at 100 V for 30 min by using a 1% agarose gel, stained with 0.5 *μ*g/mL ethidium bromide, and further visualized by UV illumination (Shanghai, China). The bands were then recorded and analyzed on a digital imaging system (Quantity One software). Levels of gene expression were expressed as the intensity of PCR products normalized that of the GAPDH in the same sample.

### 2.5. Western Blot

Frozen tissue was crushed in liquid nitrogen, homogenized in 100 *μ*L RIPA lysis buffer (50 mM Tris-Cl, pH 8.0, 150 mM NaCl, 1% (v/v) Nonidet P-40, 0.5% Sodium deoxycholate, 0.1% SDS) on ice for 30 m. Protein was quantified by using the BCA Protein Assay Kit. Samples were run on a 10% denaturing polyacrylamide gel at 80 V, transferred to a nitrocellulose membrane (Millipore) and immunoblotted with anticollagen (Santa Cruz, CA, USA) at 1 : 1000, anti-MMP1 (Santa Cruz, CA, USA) at 1 : 1000, anti-TIMP1 (Santa Cruz, CA, USA) at 1 : 1000, anti-phospho-AKT at 1 : 1000, anti-phospho-ERK (Santa Cruz, CA, USA) at 1 : 1000, and anti-*β*-actin (Santa Cruz, CA, USA) at 1 : 5000. The fluorescently labeled secondary antibodies (Santa Cruz, CA, USA) were diluted 1 : 5000 into phosphate-buffered saline—0.05% Tween (PBST)—1% ovalbumin. Antigen-antibody complexes were visualized with fluorescent labeled secondary antibodies and LI-COR Odyssey Infrared Imaging System.

### 2.6. Immunohistochemistry

Kidney tissues were fixed in 4% paraformaldehyde at room temperature for 24 h. The samples were then dehydrated with 50%, 70%, 80%, 90%, 95%, and two times of 100% alcohol, cleared with xylene, and embedded in paraffin wax. Also, 5 *μ*m sections were cut in a microtome (Leica, Germany), deparaffinized with xylene, and rehydrated in a series of ethanols. Endogenous peroxidase was blocked by 3% hydrogen peroxide in methanol for 30 m. For epitope retrieval, slides were heated in a microwave oven at 92°C for 20 m in a PBS buffer. They were then incubated overnight at 4°C with the primary antibodies anticollagen I, MMP1 at 1 : 250 (Santa Cruz, CA, USA). Sections were subsequently incubated for 1 h with biotinylated goat anti-rabbit antibody IgG and then for 30 m with Streptavidin-HRP peroxidase (Santa Cruz, CA, USA). Color reaction product was visualized by using diaminobenzidine- (DAB-) H_2_O_2_ as substrate for peroxidase. All sections were counterstained with hematoxylin, dehydrated, and covered. Incubations with phosphate-buffered saline containing 1% bovine serum albumin were used as negative controls. The positivity of immunoreactivity was evaluated semiquantitatively in a double-blind manner.

### 2.7. Statistical Analysis

Statistical analyses were performed with SPSS 15.0 software. Differences among different groups were evaluated with the student's *t*-test, and *P* < 0.05 was considered to be statistically significant.

## 3. Results

### 3.1. Blood Glucose Fluctuation Accelerate Type I Collagen Synthesis of Diabetic Mice

**Figure 2:**
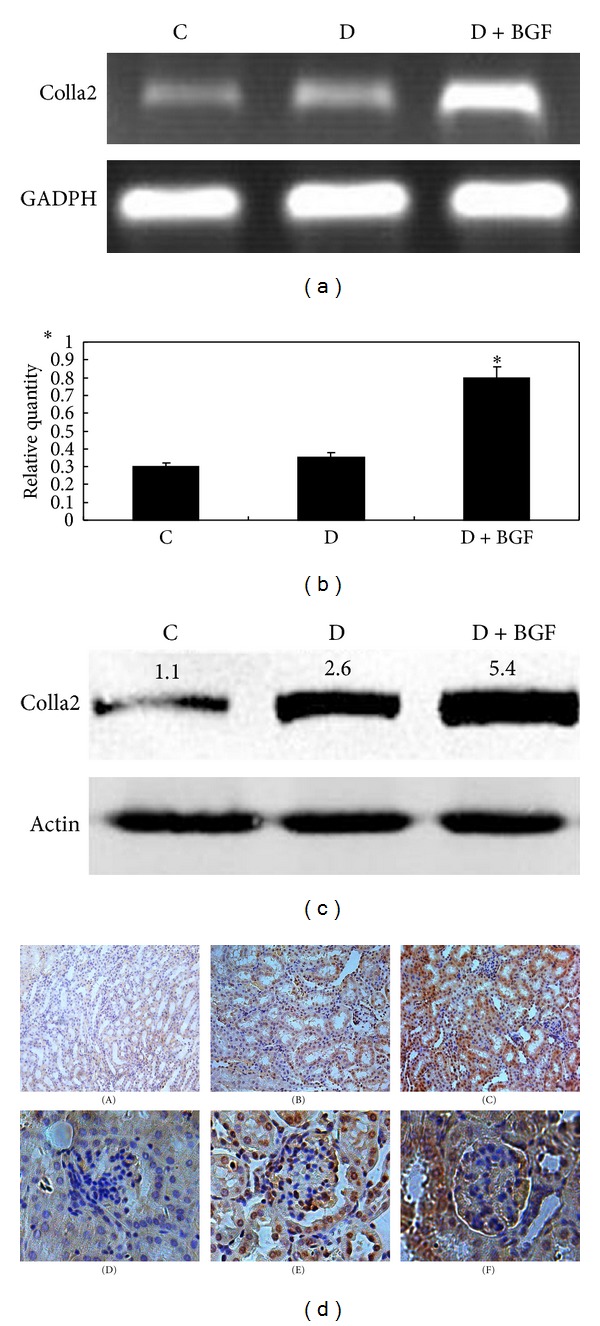
BGF treatment increased the synthesis of type I collagen. (a) Total RNA was extracted and analyzed for the expression of collagen in the kidney by RT-PCR. (b) GAPDH was used as an internal control. PCR products were semiquantified according to the ratio of type I procollagen mRNA to GAPDH mRNA. (b) Data are expressed as the mean ± SEM of three separate experiments. *P < 0.05 as compared to the control. (c) Western blot analysis depicted protein level of collagen in the kidney of mice. (d) Immunohistological staining of type I collagen in the kidney tissue of normal ((A), (D)), diabetic ((B), (E)), and BGF-treated ((C), (F)) mice. The rate of collagen positivity in the BGF-treated mice is higher than the other groups. Magnification: 200x.

To examine whether blood glucose fluctuation could affect the expression of extracellular protein, RT-PCR analysis was performed. The results showed that type I collagen expression in the kidney increased markedly after BGF treatment when compared with the only diabetic mice and controls (Figure 2(a)). The increase rate of type I collagen in the D + BGF group was 2-fold higher than in the D group (*P* < 0.05) (Figure 2(b)).

The expression of type I collagen at the protein level was determined by Western blots. Compared with the D and C groups, BGF treatments induced the maximal increase in the protein level of type I collagen (Figure 2(c)). The results were similar to those obtained by RT-PCR.

To further test the effects of BGF on the kidney of diabetic mice, we performed the immunohistochemical analysis. Collagen expression was positive in 80% of the tissue in the BGF group, 50% of diabetic tissue, and 40% of controls (Figure 2(d)). Therefore, it is obvious that collagen expression was increased significantly in the D + BGF group compared with the D and control groups.

### 3.2. Blood Glucose Fluctuation Reduce Type I Collagen Degradation of Diabetic Mice

**Figure 3:**
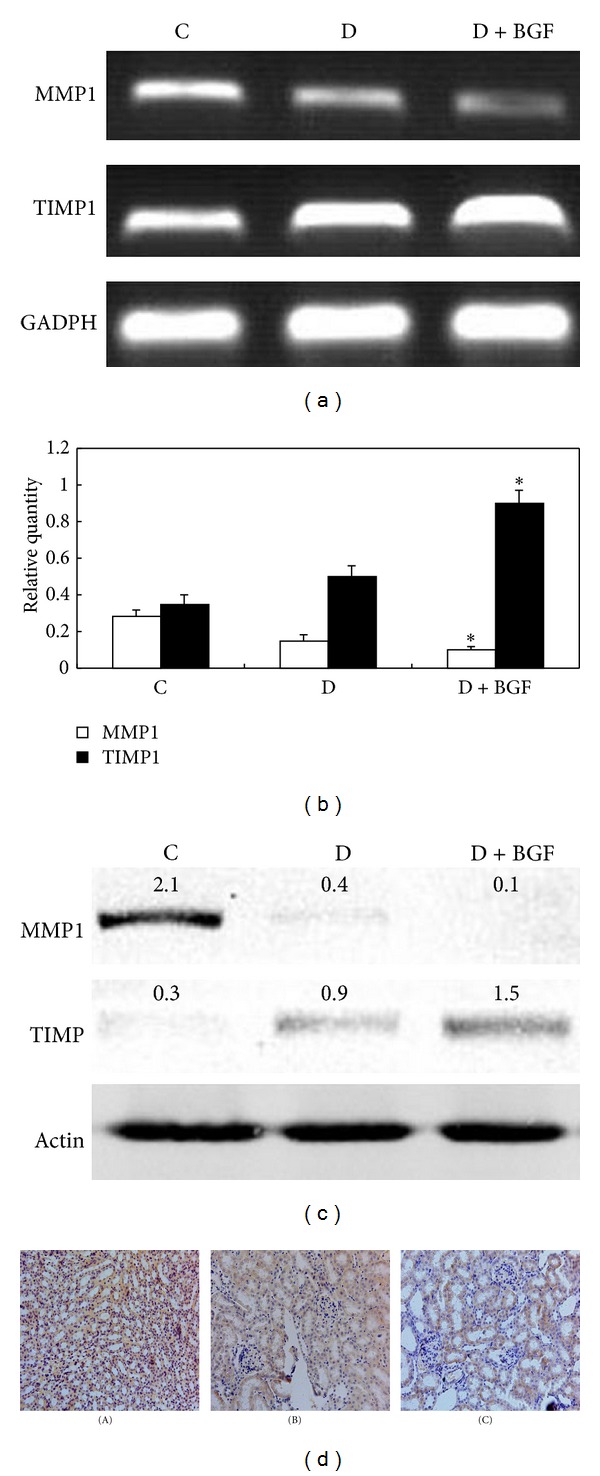
BGF treatment inhibited MMP1 and activated TIMP1. (a) MMP1 and TIMP1 expression in the kidney was evaluated by RT-PCR. (b) GAPDH was used as an internal control. PCR products were semiquantified according to the ratio of type I procollagen mRNA to GAPDH mRNA (b). Data are expressed as the mean ± SEM of three separate experiments. *P < 0.05 as compared to the control. (c) Western blot analysis depicted protein levels of MMP1 and TIMP1 in the kidney of mice. (d) Immunohistological staining of MMP1 in the kidney of mice. The rate of MMP1 positivity in the BGF-treated mice (C) was lower than the normal (A) and diabetic (B) mice. Magnification: 200x.

RT-PCR results showed that MMP1 mRNA levels were significantly decreased in the D + BGF group (Figure 3(a)). Although the mRNA level of MMP1 in the D group was also lower than the control, more decrease was observed in the D + BGF group than in only diabetic group (*P* < 0.05) (Figure 3(b)). In contrast, TIMP1 mRNA expression in the D + BGF group was elevated markedly compared with the D and C groups (Figures 3(a) and 3(b)). The proteins of MMP1 and TIMP1 were also evaluated by Western blot analysis. As expected, the protein production of MMP1 in the D + BGF group was markedly inhibited compared with the control and diabetic mice, while the TIMP1 level was higher than the other groups (Figure 3(c)).

A decrease of MMP1 positivity was observed simultaneously in the tissues of the BGF group by immunohistological staining. The MMP1 positive staining presents in 30% of tissue in BGF group, while the control mice showed 80% MMP1 positivity in the kidney (Figure 3(d)). Thus, consistent with the PCR and Western blotting analyses, immunohistological staining supported that collagen synthesis was rather increased while collagen degradation was quite reduced in the D + BGF group.

### 3.3. ERK Was Activated by Blood Glucose Fluctuation of Diabetic Mice

**Figure 4:**
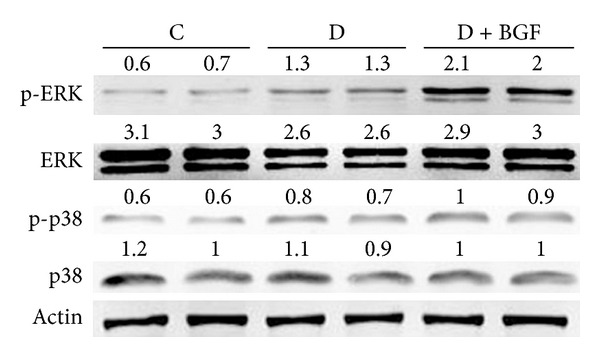
BGF treatment enhanced ERK1/2 phosphorylation in the kidney of mice. The p38 activity was not sensitive to BGF treatment. Whole cell lysates were collected and subjected to Western blot analysis for phosphorylated ERK1/2, p38 and total ERK 1/2, p38.

To determine whether blood glucose fluctuation might increase collagen synthesis in the kidney by activating extracellular signal-regulated kinase/mitogen-activated protein kinase (ERK/MAPK) signaling pathway, the effect of blood glucose fluctuation on the phosphorylation of ERK1/2 was examined. As seen in Figure 4, blood glucose fluctuation markedly induced MAPK/ERK activation. The activation of ERK was increased by twofold when compared with the only D group. Although the diabetes could also elevate the level of phospho-ERK1/2, the increase rate is far less than that of BGF treatment. Western blot analyses showed that the total protein levels of ERK in the BGF group were the same as the D and C groups. However, p38 showed little sensitivity to BGF treatment in the mice. The level of phosphor-p38 was almost unchanged in the control, diabetic, and the BGF mice.

### 3.4. Blood Glucose Fluctuation Increased TGF-*β* and p-Smad2 Expression of Diabetic Mice

**Figure 5:**
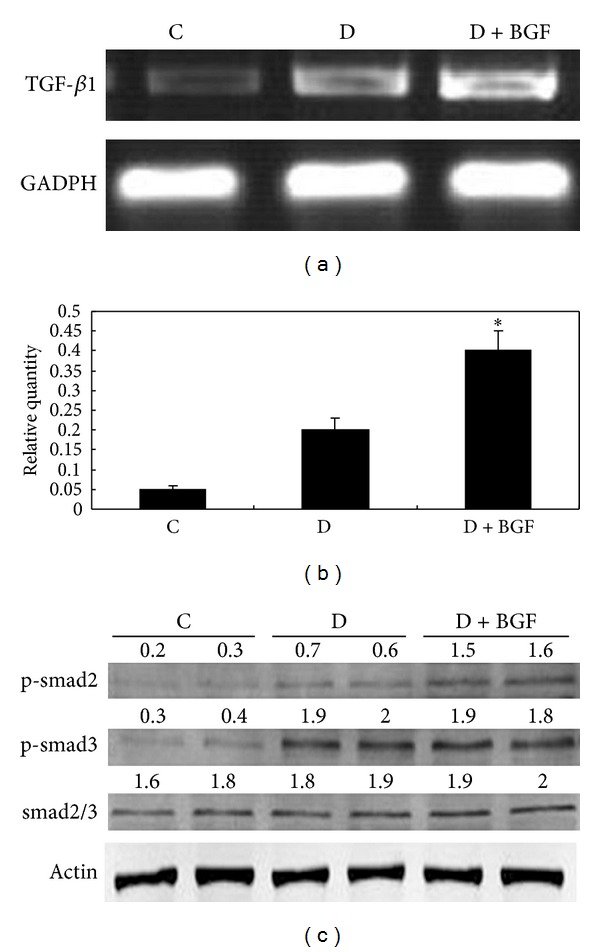
BGF treatment upregulated TGF-*β*1 expression and activated TGF-*β*1-induced smad signaling. (a) TGF-*β*1 expression in the kidney of mice was evaluated by RT-PCR. (b) Data are expressed as the mean ± SEM of three separate experiments. *P < 0.05 as compared to the control. (c) The phosphorylated forms of smad2 and smad3 were analyzed by Western blot, using either rabbit anti-p-smad2 or rabbit anti-p-smad3 and total rabbit anti-smad2/3 antibody.

Blood fluctuation significantly increased TFG-*β*1 mRNA expression compared with the untreated mice (Figure 5(a)). The TGF-*β*1 levels in the kidney treated with BGF for eight weeks were 8 times the control (Figure 5(b)). Compared with the only diabetic mice, the TGF-*β*1 mRNA expression of D + BGF mice increased twofold (*P* < 0.05). So, blood glucose fluctuation caused more increase of TGF-*β* level than only diabetes.

To elucidate whether smad activity was increased by the upregulated expression of TGF-*β*1, the detection of phosphorylation of smad2 and smad3 was carried out.  Figure 5(c) clearly showed that the expression of p-smad2 in the kidney of BGF-treated mice was markedly increased compared with the controls. The elevation of p-smad2 was evident as a 2-fold increase in the kidneys of diabetic mice. The expression of p-smad3 also demonstrated a marked increase compared with controls. However, p-smad3 was similar between the diabetic mice and BGF-treated mice. These results showed that the increase of p-smad2 induced by BGF was more strongly than that of diabetes.

### 3.5. AKT Was Not Affected by Blood Glucose Fluctuation of Diabetic Mice

**Figure 6:**
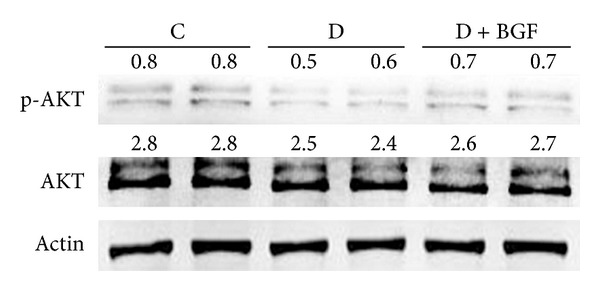
The activity of PIP3/AKT signal pathway was unchanged after BGF treatment. The phosphorylated and total AKT levels were evaluated by Western blot analysis.

PKB/AKT signaling molecules have been reported to be activated by TGF-*β*1 and play a role in renal fibrosis during diabetic nephropathy. To determine the effects of blood glucose fluctuation on the activation of this signal pathway, phosphospecific antibody to AKT was used in Western blot. As shown in Figure 6, the level of AKT phosphorylation in the kidneys of BGF-treated mice is similar with the control. No differences in AKT phosphorylation among the BGF-treated group, consistent hyperglycemia group and controls, were noted. 

## 4. Discussion

Previous study indicates that repetitive postprandial fluctuation in glucose concentration evokes monocyte adhesion to endothelial cells, enhances endothelial cell apoptosis, and accelerates atherosclerosis that was worse than that induced by stable hyperglycemia *in vivo* [11–13]. Glucose fluctuations during postprandial periods also exhibited a more specific triggering effect on oxidative stress than chronic sustained hyperglycemia [14]. Moreover, BGF was demonstrated to damage skin collagen metabolism in mouse skin [15]. In this study, we find that treatments with BGF resulted in increased expression and deposition of type I collagen and decreased collagen degradation when compared to the stable hyperglycemia mice. The overproduction of the interstitial matrix components such as type I and type III collagen and fibronectin is thought to be the most important event in naturally occurring renal fibrosis [8]. Decreased MMPs and increased expression of TIMP1 participate in the excessive collagen deposit during the evolution of experimental interstitial renal fibrosis [16]. Thus, our data indicated that BGF may be related with the development of renal fibrosis in diabetic mice. BGF seems to produce more deleterious effects on accelerating of renal fibrosis of diabetic mice than only consistent hyperglycemia. Therefore, besides the traditional therapeutic methods for DN, more attention should be payed to inhibiting BGF in the diabetes.

Renal fibrosis is characterized by the increased accumulation of extracellular matrix (ECM) within renal tissue. An abnormal matrix deposition may be due to increased synthesis or decreased degradation of ECM. Our present study demonstrated that BGF not only induces the expression of ECM, but also inhibits its degradation by inhibiting matrix metalloproteases (MMPs) and activating the tissue inhibitor of metalloproteinases (TIMPs), which supported the importance of BGF in the progress of renal fibrosis in the diabetes. A previous study demonstrated that short-term peaks in glucose increased the production of collagen type IV, fibronectin, MMP2, MMP9, and TGF-1 in human renal cortical fibroblasts [17]. Thus, they concluded that exposure to fluctuating glucose concentrations increases renal interstitial fibrosis *in vitro*, which is consistent with our results *in vivo*.

However, the mechanisms by which BGF affects renal fibrosis remain unclear. The MAPK pathways are well characterized in regulating ECM expression. A previous report showed that the single blockade of p38 MAPK after the emergence of established fibrosis is effective to reduce subsequent renal fibrosis in the model of unilateral ureteral obstruction [18]. We had some indication, in renal fibrosis, that MAPK pathway may have some roles in promoting BGF-induced abnormal deposition of ECM. Western blot analysis indicated that the level of the phosphorylation of ERK1/2 increased significantly in comparison with the controls and the stable hyperglycemia group. Consistent with the increased collagen synthesis, treatments with only hyperglycemia also cause the activation of ERK pathway compared to controls, but the effect was less than that of BGF treatment. The positive relationship between pERK and collagen overproduction indicated that it is possible that BGF accelerated renal fibrosis by activating the ERK pathway. However, our results showed that the role of p38 in the BGF-induced renal fibrosis can be neglected. It is regrettable that we could not detect the p-JNK expression. We assumed that the role of JNK signaling in the process of BGF-induced renal fibrosis may be minimal.

A previous study has demonstrated that TGF-*β* was a key mediator of hyperglycemia-induced increase in ECM accumulation [19]. In addition, blocking TGF-*β*'s function has been described to suppress excess ECM accumulation and glomerulosclerosis [20]. Similar with the previous results, we found that hyperglycemia indeed increased the expression of TGF-*β*1 when compared to the controls. However, the level of TGF-*β*1 in the D + BGF group was more than that of stable hyperglycemia group, indicating that BGF can accelerate the accumulation of ECM than only diabetes. Several data showed that both smad2 and smad3 mediate the signal transduction from TGF-*β*1 [21, 22]. Thus, we detected the phosphorylation of smad2 and smad3 by Western blot. We observed BGF markedly increased the levels of p-samd2 when compared the hyperglycemia mice. Our results indicated that BGF-accelerated renal fibrosis also appears to be related with the activation of TGF-*β*/smad signal pathway. However, p-smad3 showed the similar increases in the BGF-treated and only diabetic mice compared with the controls. Smad7 is an inhibitory smad that functions to block smad2/3 activation by degrading the T*β*RI and smads [23]. Also, it can inhibit NF-*κ*B-driven inflammatory response by inducing I*κ*B*α* [24]. Kidney-targeting smad7 gene transfer inhibits renal TGF-*β*/MAD homologue (smad) and nuclear factor *κ*B (NF-*κ*B) signalling pathways and improves diabetic nephropathy in mice [25]. Thus, expression of smad7 and NF-*κ*B in response to blood glucose fluctuations should be performed in our further experiments. Further studies are also needed to determine the exact relationship between ERK/MAPK and TGF-*β*/smad signaling in the development and progression of diabetic renal fibrosis.

Another previous study showed that TGF-*β*-mediated PKB/Akt activation may be important in renal fibrosis during diabetic nephropathy [26]. The activated PI3-K/Akt was found to participate in regulation of HSC migration, proliferation, collagen secretion, and adhesion. However, we could not find any elevation of phosphorylated-AKT in the hyperglycemia mice and BGF-treated mice compared with the controls. Thus, these data showed that Akt may not serve as a mediator of BGF induced renal fibrosis during the pathogenesis of DN. 

In summary, we have shown in this present study that BGF could accelerate the progress of renal fibrosis under high glucose conditions by increasing extracellular matrix protein synthesis and inhibiting its degradation. Furthermore, we demonstrate that both ERK/MAPK and TGF-*β*/smad signaling pathways seem to play a role in the kidney fibrosis of diabetic mice accelerated by blood glucose fluctuation. The data suggest that inhibition of fluctuations in glucose concentrations may be of potential benefit in preventing the renal fibrosis in the diabetic patients. However, diabetic nephropathy as well as interstitial fibrosis is a late occurrence during the development of diabetes. So, it is worthwhile to make a long-term animal model to confirm the results in this study.

## Figures and Tables

**Table 1:** Primers for PCR.

Gene	Primer sequence-forward	Primer sequence-reverse
GAPDH	GGAGACAACCTGGTCCTCAG	ACCCAGAAGACTGTGGATGG
Col I(*α*2)	CTTGTGGCTTCTGACTATCT	AGGAAAATGAGGCTGTTA
MMP1	TTCTGAAACCCTGAGTGC	AAGCCTGGATGCGATTA
TIMP1	AGTGGGGTCTGTGAGGT	CAAAAGAGGGAGTGCTG
TGF-*β*	CGGTGCTCGCTTTGTA	GCCACTCAGGCGTATC
